# CSPG4 Expression in GIST Is Associated with Better Prognosis and Strong Cytotoxic Immune Response

**DOI:** 10.3390/cancers14051306

**Published:** 2022-03-03

**Authors:** Alexandre de Nonneville, Pascal Finetti, Maelle Picard, Audrey Monneur, Maria Abbondanza Pantaleo, Annalisa Astolfi, Jerzy Ostrowski, Daniel Birnbaum, Emilie Mamessier, François Bertucci

**Affiliations:** 1 Predictive Oncology Laboratory, Equipe Labellisée Ligue Nationale Contre Le Cancer, Centre de Recherche en Cancérologie de Marseille (CRCM), Institut Paoli-Calmettes, Inserm UMR1068, CNRS UMR7258, Aix-Marseille University, 13009 Marseille, France; denonnevillea@ipc.unicancer.fr (A.d.N.); finettip@ipc.unicancer.fr (P.F.); maelle.picard@inserm.fr (M.P.); daniel.birnbaum@inserm.fr (D.B.); emilie.mamessier@inserm.fr (E.M.); 2 Department of Medical Oncology, Institut Paoli-Calmettes, Aix-Marseille University, CNRS, INSERM, 13009 Marseille, France; monneura@ipc.unicancer.fr; 3 Department of Specialized, Experimental and Diagnostic Medicine, Sant’Orsola-Malpighi Hospital, University of Bologna, 40138 Bologna, Italy; maria.pantaleo@unibo.it (M.A.P.); annalisa.astolfi@unibo.it (A.A.); 4 Department of Gastroenterology, Hepatology and Clinical Oncology, Medical Center of Postgraduate Education, 01-813 Warsaw, Poland; jerzy.ostrowski@pib-nio.pl; 5 Department of Genetics, Maria Sklodowska-Curie National Institute of Oncology, 02-781 Warsaw, Poland

**Keywords:** CSPG4, gene expression, GIST, immune response, prognosis, CAR-CIKs, NK cells

## Abstract

**Simple Summary:**

Gastrointestinal stromal tumors (GIST) are the most frequent sarcomas of the gastrointestinal tract. Identification of novel prognostic and/or therapeutic targets is a major issue to overcome tyrosine kinase inhibitors resistances. CSPG4, a cell surface proteoglycan, emerged as a potential therapeutic target for immune therapy in different cancers, including sarcomas. CSPG4 expression has never been studied in GIST. In this work we analyzed CSPG4 mRNA expression in a large series of clinical GIST samples given the scarcity of disease (*n* = 309 patients). We find that high CSPG4 expression is independently associated with disease-free survival, and with an immune landscape favorable to induce strong cytotoxic immune response after NK cell stimulation. Our results suggest the potential value of CSPG4-specific chimeric antigen receptor-redirected cytokine-induced killer lymphocytes treatment in GIST, notably “CSPG4-high” tumors, and calls for preclinical validation, drug testing in vivo, then in clinical trials.

**Abstract:**

The treatment of gastrointestinal stromal tumors (GIST) must be improved through the development of more reliable prognostic factors and of therapies able to overcome imatinib resistance. The immune system represents an attractive tool. CSPG4, a cell surface proteoglycan, emerged as a potential therapeutic target for immune therapy in different cancers, including cell therapy based on CSPG4-specific chimeric antigen receptor (CAR)-redirected cytokine-induced killer lymphocytes (CSPG4-CAR.CIKs) in sarcomas. CSPG4 expression has never been studied in GIST. We analyzed CSPG4 mRNA expression data of 309 clinical GIST samples profiled using DNA microarrays and searched for correlations with clinicopathological and immune features. CSPG4 expression, higher in tumors than normal digestive tissues, was heterogeneous across tumors. High expression was associated with AFIP low-risk, gastric site, and localized stage, and independently with longer postoperative disease-free survival (DFS) in localized stage. The correlations between CSPG4 expression and immune signatures highlighted a higher anti-tumor immune response in “CSPG4-high” tumors, relying on both the adaptive and innate immune system, in which the boost of NK cells by CSPG4-CAR.CIKs might be instrumental, eventually combined with immune checkpoint inhibitors. In conclusion, high CSPG4 expression in GIST is associated with better DFS and offers an immune environment favorable to a vulnerability to CAR.CIKs.

## 1. Introduction

Gastrointestinal stromal tumors (GIST) are the most frequent sarcomas of the gastrointestinal tract [1,2]. They remain an exemplary model for targeted therapies within solid tumors: the presence of KIT or PDGFRA activating mutations (~85% of cases) [3,4] leads to high efficiency of the tyrosine kinase inhibitors (imatinib, sunitinib, regorafenib, and avapritinib) [5,6] currently marketed. In advanced stages, first-line imatinib increases both the response rate (70% vs. <10% with chemotherapy) and the median overall survival (76 months in the recent BFR14 clinical trial^6^ vs. <10 months with chemotherapy). However, primary or secondary resistance occurs in all cases and a critical goal is to develop new therapies able to overcome it. Adjuvant imatinib results in lower relapse rates [7,8] and increases overall survival [8] in localized stages treated by surgery [2]; it is currently recommended for patients at intermediate or high risk of metastatic relapse, according to the AFIP classification [2,9]. Nevertheless, classifications based on clinicopathological features, such as AFIP, remain imperfect and need to be improved [5,10,11,12,13]. Identification of novel prognostic and/or therapeutic targets is a major issue.

The role of immunotherapy in sarcomas is growing fast with no current standard of care. If our current understanding of the immune response in GIST remains limited, several data suggested that the exploitation of immune system may be clinically interesting [14]. The presence of tumor-infiltrating immune cells such as macrophages, CD8+ T-cells, T-reg, and NK-cells has been described in clinical samples [15,16,17,18] and associated with prognosis [17], imatinib response [15,19,20], and potential vulnerability to immune checkpoint inhibitors [21]. The oncolytic action of imatinib also partly relies on indirect effects of immune cell actions, notably CD8+ T-cells [15] and NK-cells [22]. Consistently, the concurrent CTLA-4 blockade increased the efficacy of imatinib in mouse GISTs through activation of IFNγ-producing CD8+ T-cells [15]. We have reported a correlation between *PDL1* expression, immune-related parameters, and prognosis in imatinib-naïve patients with localized GIST [23]. Altogether, these pre-clinical data suggested a role of the immune system in the treatment of GIST. Several potential immunotherapeutic strategies have been tested or are under investigation in GIST clinical trials (PMID: 34298737). One of them is cellular therapy: chimeric antigen receptor (CAR) T-cells with anti-KIT activity showed antitumor effects in vitro and in vivo, notably against imatinib-resistant cells [24]. During the last years, CSPG4 was described as another potential target of cellular immunotherapy in cancers [25], and more recently soft tissue sarcomas, including GIST [26].

CSPG4, a cell surface proteoglycan, displays overexpression in certain human cancers, low expression in normal tissues, and roles in tumor growth and dissemination [25]. Although widely unexplored, GSPG4 also influences activation, maturation, proliferation, and migration of different immune cell subsets [25], suggesting likely interaction with immunotherapy efficiency. Recently, CSPG4-specific chimeric antigen receptor (CAR)-redirected cytokine-induced killer lymphocytes (CSPG4-CAR.CIKs) effectively targeted multiple soft tissue sarcomas (STS) histotypes in vitro and in vivo [26]. The series of tested cell lines included several GIST cell lines derived from patients in relapse after conventional treatment: all cell lines showed CSPG4 expression at the cell surface, and tumor elimination was strictly dependent on the expression level on tumor cells.

To our knowledge, CSPG4 expression has not been studied in GIST clinical samples. Here, we analyzed CSPG4 expression in 309 GIST samples and searched for correlations with both the clinicopathological features including clinical outcome and the tumor immune landscape. We show that expression is heterogeneous, high GSPG4 is a favorable independent prognostic factor and is associated with an immune profile suggesting potential vulnerability to cellular immunotherapy.

## 2. Materials and Methods

### 2.1. Tumor Samples

Our data set included clinicopathological and gene expression data of clinical GIST samples from 15 public data sets that we collected from the National Center for Biotechnology Information (NCBI)/Genbank GEO, ArrayExpress databases, and authors’ websites (Table S1). The samples had been profiled using commercial or homemade whole-genome DNA microarrays. The pooled data set contained 309 clinical samples. The study was approved by our institutional board. We also analyzed cancer cell lines data from the Dependency Map (DepMap) portal (https://depmap.org/portal; accessed on 14 December 2021) to compare the RPPA-based protein vs. RNA-seq-based mRNA expression of CSPG4.

### 2.2. Gene Expression Data Analysis

A pre-analytic processing of data was done. In a first step, we normalized each data set separately: a quantile normalization was applied to the already processed non-Affymetrix data, and Robust Multichip Average (RMA) with non-parametric quantile algorithm to the raw Affymetrix data. That was done in R using Bioconductor and associated packages. In a second step, we mapped hybridization probes represented across the different technological platforms, and when multiple probes mapped to the same GeneID, we kept the most variant probe in a given data set.

Next, we corrected the 15 studies for batch effects using z-score normalization. Briefly, for each *CSPG4* expression value in each study separately, the value was transformed by subtracting the mean of the gene in that dataset divided by its standard deviation in the GIST samples. Analysis was done by using binary values using the median expression level of the whole series as a cut-off. Because CSPG4 is the target of cellular immunotherapy, we searched for correlations of its expression in tumors with several immune variables. In each data set separately, several immunity-related multigene classifiers were applied to each tumor: the 24 Bindea’s innate and adaptative immune cell subpopulations [27], the Immunologic Constant of Rejection (ICR) classifier [28], and metagenes associated representative of T-cell-inflamed signature (TIS) [29], of tertiary lymphoid structures (TLS) signature [30], of cytolytic activity score [31], of IFNα and IFNγ pathways activation score [32], the antigen processing machinery (APM) score [33], and ESTIMATE scores (Immune infiltration, Stromal infiltration, Tumor purity) [34].

### 2.3. Statistical Analyses

The continuous variables are presented using median and range, whereas the discrete values are presented using number and percentage. The correlations between *CSPG4* expression-based groups and clinicopathological variables were calculated with the Student’s *t*-test or Fisher’s exact test when appropriate. Since the delay of relapse and follow-up were not available in seven data sets, we used as primary endpoint the occurrence of disease relapse or death during follow-up. The prognostic analyses for disease-free survival (DFS) were done using a logistic regression based on the lm function (R’s statistical package), the significance of which was estimated by specifying a binomial family for model with a logit link. Several variables were tested in univariate analysis: the *CSPG4* expression status (“CSPG4-low”, “CSPG4-high”), the patients’ age and sex (male, female), the tumor location (gastric, small intestine, other) and mutational status (*KIT*, *PDGFRA*, wild-type), and finally the AFIP risk (high-risk, intermediate/low-risk). All variables with a *p*-value inferior to 5% in univariate analysis were included in the multivariate analysis. When the time to follow-up was available, the 5-year DFS was estimated using the Kaplan-Meier curves; and curves were compared using the log-rank test. A logistic regression analysis using the lm function tested the correlations of molecular variables with “CSPG4-high” vs. “CSPG4-low” groups (R’s package). All statistical tests were two-sided and the significance threshold was 5%. All analyses were done with the survival package (version 2.43) from R software (version 3.5.2).

## 3. Results

### 3.1. Patients’ Characteristics and CSPG4 Expression

A total of 309 GIST samples was available for analysis. Their characteristics are summarized in Table 1. The median patients’ age was 61 years (range, 8–87), the gender was male in 57% of cases, and the anatomical location was the stomach in 74% of cases, followed by the small intestine in 18%. Regarding the mutational status, available in 275 cases, the mutations most often represented concerned *KIT*, mainly in exon 11, then *PDGFRA*, mainly in exon 18. A wild-type *KIT* and *PDGFRA* status (WT) was found in 16% of cases (WT). The disease extension stage was localized in most of cases (83%) and advanced (locally advanced or metastatic) in 17%. The relapse risk, defined according to the AFIP classification, was low-risk in 53% of cases, intermediate-risk in 19%, and high-risk in 28%.

*CSPG4* mRNA expression varied among the 309 tumors with a range of intensities over 8 units in log_2_ scale (Figure S1A), suggesting a heterogeneous expression across clinical GIST samples. Of note, the *CSPG4* mRNA expression was strongly correlated with protein expression in a series of 369 cancer cell lines (Figure S1B). Furthermore, analysis using the IST Online tool (http://ist.medisapiens.com; accessed on 7 June 2021) showed higher mRNA CSPG4 expression in GIST samples (*N* = 77) than in digestive tract normal tissues (*N* = 144) (Figure S1C).

### 3.2. CSPG4 Expression and Correlations with Clinicopathological Features

We searched for correlations between *CSPG4* expression (high vs. low) and clinicopathological features (Table 1). There was no significant correlation with patients’ sex and age. A trend for correlation existed with the mutational status (*p* = 0.063), with more *PDGFRA* mutations in “CSPG4-high” tumors and more WT status in “CSPG4-low” tumors. Significant correlations were found with prognostic features: AFIP classification, with more low-risk in “CSPG4-high” tumors and more intermediate- and high-risk in “CSPG4-low” tumors (*p* = 3.83 × 10^−4^); tumor site, with more gastric site in “CSPG4-high” tumors and more small intestine and other sites in “CSPG4-low” tumors (*p* = 4 × 10^−6^); and disease stage, with more localized diseases in “CSPG4-high” tumors and more advanced disease in “CSPG4-low” tumors (*p* = 2.83 × 10^−3^).

### 3.3. CSPG4 Expression and Correlation with Disease Relapse

A total of 161 out of 309 patients fulfilled the following criteria—localized tumors, treated with primary complete surgery, without adjuvant imatinib, and with available information regarding an eventual disease relapse—and were thus included in the prognostic analysis. Their characteristics are summarized in Table S2. Thirty-two experienced a relapse and 129 did not. As shown in Table 1, only 8% of patients in the “CSPG4-high” group experienced a relapse vs. 39% in the “CSPG4-low” group (*p* = 3.79 × 10^−6^). In univariate analysis (Table 2), AFIP high-risk (*p* = 2.82 × 10^−10^) class was associated with a higher risk of relapse when compared with low/intermediate-risk class, whereas patients’ age, sex, and mutational status were not significant.

In multivariate analysis, CSPG4 expression-based group (*p* = 5.12 × 10^−4^; logit test), and AFIP high-risk (*p* = 6.93 × 10^−9^) class remained significant, suggesting independent prognostic value (Table 2). The stratification of patients according to the CSPG4 group and the AFIP risk identified four subgroups with different relapse rates. For example, *CSG4* expression affected the clinical outcome of AFIP high-risk patients: with an Odds Ratio (OR) for relapse of 0.85 (95%CI 0.78–0.92) in CSPG4-high vs. CSPG4-low patients. Similarly, among the AFIP low/intermediate-risk patients, the Odds Ratio (OR) for relapse was 0.74 (95%CI 0.56–0.99) in CSPG4-high vs. CSPG4-low patients.

**Figure 1:**
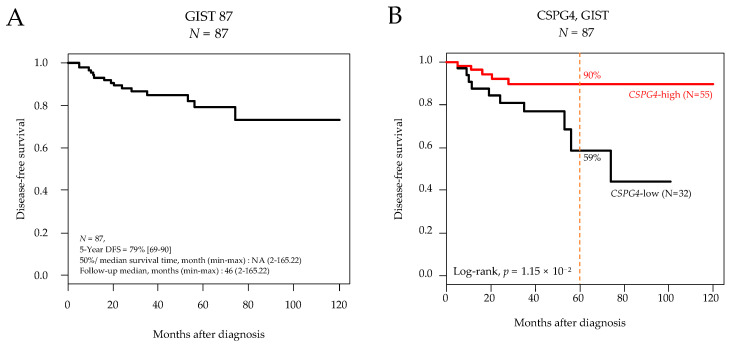
Disease-free survival in patients with localized GIST after surgery. (**A**) Kaplan-Meier DFS curves in the 87 informative patients for DFS. (**B**) Similar to (**A**), but according to CSPG4 expression (low and high). The *p*-value is for the log-rank test.

Eighty-seven patients were informative regarding the follow-up duration. With a median follow-up of 42 months after surgery, the 5-year DFS was 79% (95%CI 69–90) in the whole series (Figure 1A) and was different according to CSPG4 expression with 90% 5-year DFS (95%CI 81–99) in the “CSPG4-high” group vs. 59% (95%CI 38–90) in the “CSPG4-low” group (*p* = 1.15 × 10^−2^, log-rank test; Figure 1B).

### 3.4. CSPG4 Expression and Correlation with Imatinib Sensitivity

**Figure 2:**
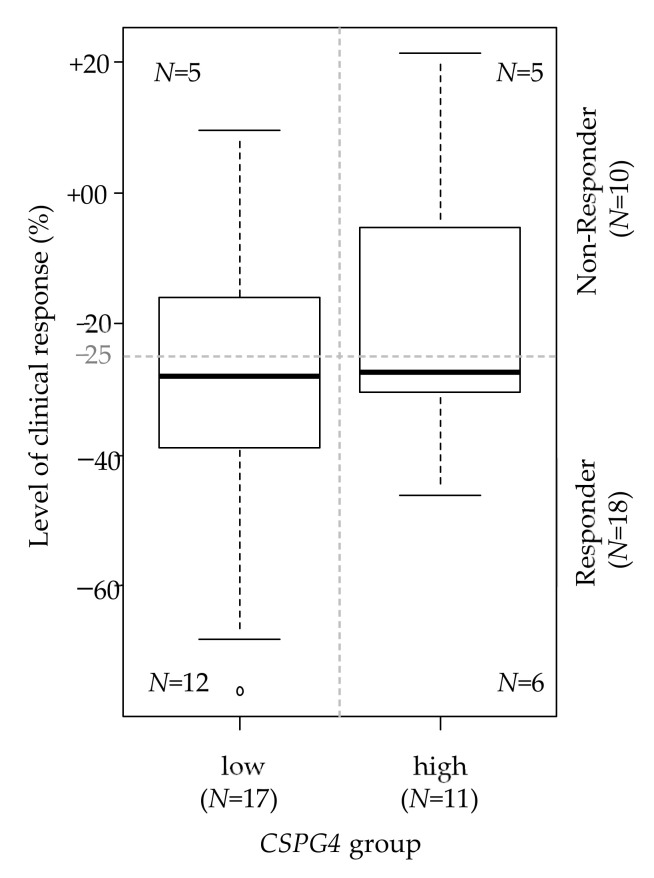
Correlation between CSPG4 expression and the response to imatinib. Correlation between the two CSPG4-based groups (high and low; *N* = 28) and the level of clinical response to neoadjuvant imatinib assessed as a continuous variable (box plot). The horizontal dashed line indicates the cut-off of tumor shrinkage that defines the responder status. The figures within the box plot indicate the number of patients in each of the four categories.

We then searched for a correlation between CSPG4 expression and the sensitivity to imatinib, the standard first-line treatment. We analyzed 28 pre-treatment tumor samples from patients treated in the RTOG0132 phase II trial with neoadjuvant imatinib during 8 to 12 weeks for an advanced primary/recurrent GIST and for whom the RECIST clinical response was available [35]. The degree of tumor shrinkage ranged from –76% to +21%. Ten patients were defined as “Non-Responders” (36%) and 18 as “Responders” (64%). As expected, given the advanced stage of disease and the correlation between stage and CSPG4 expression, more patients were classified as CSPG4-low (*N* = 17) than CSPG4-high (*N* = 11). We found more “Responders” in the “CSPG4-low” group than in the “CSPG4-high” (71% vs. 55%), but the difference was not significant, likely because of the small number of patients (*p* = 0.444, Fisher’s exact test; Odds Ratio = 0.51 (95%CI 0.08–3.21)). As a continuous value, the tumor shrinkage was greater in the “CSPG4-low” group than the “CSPG4-high” group (mean −30% vs. −18%, *p* = 0.159; Figure 2).

### 3.5. CSPG4 Expression and Correlations with Immune Features

**Figure 3:**
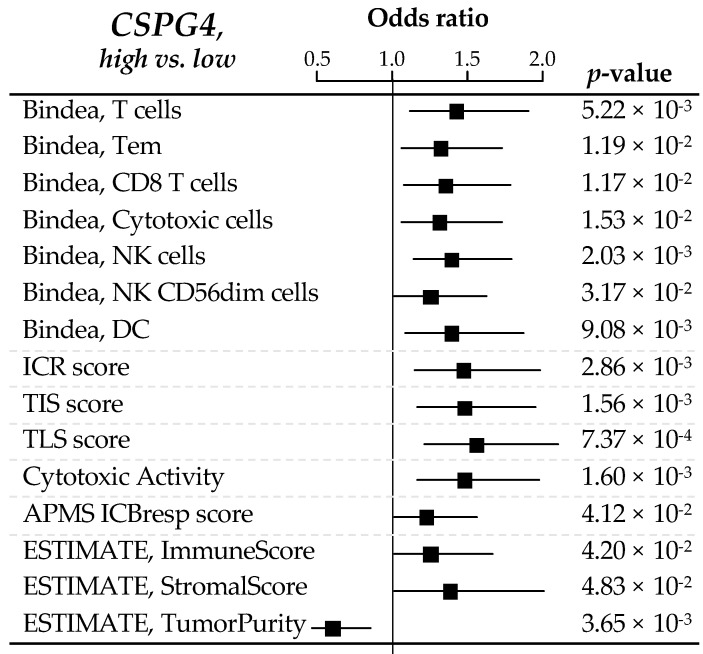
Correlations between *CSPG4* expression and immune features. Forrest plot of correlations between CSPG4-high (**left**) and -low (**right**) expression and immune features, including the composition and functional orientation of the immune infiltrate according to the Bindea’s immunome, the Immunologic Constant of Rejection signature, T cell-inflamed signature (TIS) and tertiary lymphoid structures (TLS) enrichment signatures, the immune cytolytic activity score, the antigen processing/presentation machinery (APM) score, and ESTIMATE tool analysis for immune or stromal infiltrating cells. The *p*-values are for the logit link test.

We searched for correlations between CSPG4 expression and immunity-related variables in the 309 clinical GIST samples. To determine if there was a difference in the quantity of immune or stromal cells infiltrating “CSPG4-high” and “CSPG4-low” tumors, we used the ESTIMATE tool, which provides scores for tumor purity, the level of stromal cells present, and the infiltration level of immune cells in tumor tissues, based on expression data. “CSPG4-high” GISTs displayed higher scores for immune and stromal signatures (respectively, *p* = 4.20 × 10^−2^ and *p* = 4.83 × 10^−2^), while “CSPG4-low” tumors showed higher tumor purity (*p* = 3.65 × 10^−3^). This identified “CSPG4-high” tumors as more infiltrated by immune cells than “CSPG4-low” tumors (Figure 3).

We next looked at the composition and functional orientation of this immune infiltrate in “CSPG4-high” vs. “CSPG4-low” tumors. The analysis of the 24 immune cell types defined as the immunome [27] revealed that “CSPG4-high” tumors differentiated themselves from “CSPG4-low” tumors by a higher infiltrate in dendritic cells (DC, *p* = 0.009), in T lymphocytes (T cells: *p* = 5.22 × 10^−3^; Tem: *p* = 1.19 x 10^−2^; CD8 T cells: *p* = 1.17 × 10^−2^) and more specifically with cytotoxic cells subsets (Cytotoxic cells: *p* = 1.53 × 10^−2^; NK cells, *p* = 2.03 × 10^−3^; NK CD56dim cells: *p* = 3.17 × 10^−2^).

Additional immune functional signatures reinforced this observation, showing higher Immunologic Constant of Rejection (ICR) score (*p* = 2.86 × 10^−3^) [36] in “CSPG4-high” tumors, enrichment in T cell-inflamed signature (TIS) (*p* = 1.56 × 10^−3^) [29] and tertiary lymphoid structures (TLS) signature (*p* = 7.37 × 10^−4^) [30], coherently with a higher immune cytolytic activity score (*p* = 1.60 × 10^−3^) [31] from both innate and adaptive immune effector cells. This was also in line with enhanced activation of the IFNα and IFNγ activation pathways, two cytokines with anti-tumor activity [32], in “CSPG4-high” tumors (respectively, *p* = 6.46 × 10^−3^ and *p* = 3.23 × 10^−3^). Antigen processing/presentation machinery (APM) score [33] was also significantly enhanced in “CSPG4-high” tumors (*p* = 4.12 × 10^−2^).

## 4. Discussion

We analyzed *CSPG4* expression in 309 GIST clinical samples. We report that high expression is an independent, favorable prognostic factor for disease relapse and is associated with cytotoxic immune response. To our knowledge, this is the first study analyzing the expression of this new potential target for immune therapy in GIST.

Our preliminary analysis of more than 350 cancer cell lines revealed a strong correlation between mRNA and protein expression levels, allowing us to base our study on *CSPG4* mRNA expression. Such an approach allowed us to avoid the limitations of immunohistochemistry (specificity and reproducibility of available antibodies, definition of positivity cut-off, etc.) while providing opportunities to work on a relatively large series of samples and to search for associations with the expression of other multigene signatures.

As already observed in many cancers, we confirmed higher expression in GIST tumor samples than in normal digestive tissues. *CSPG4* expression in GIST was heterogeneous, making possible to search for correlations with clinicopathological variables. High expression was associated with favorable prognostic variables: low-risk according to AFIP classification, gastric site, and localized extension stage at diagnosis. In the literature, other immune tumor features have been found to be associated with a more favorable AFIP class, such as high NK cells infiltration, low T-regs infiltration [17], and high PDL1 expression [23], suggesting that GIST cell-intrinsic features may influence the immune microenvironment. *CSPG4* expression was higher in samples from patients without disease relapse than in samples from patients with relapse. This favorable prognostic value was conserved in multivariate analysis, as was conserved the prognostic value of AFIP classification. In addition, we showed that “CSPG4-low” patients seemed more sensitive to neoadjuvant imatinib than “CSPG4-high” patients; however, the results were non-significant, likely because of the small number of patients, which calls for an analysis of larger series of patients. The prognostic value of high *CSPG4* expression has already been reported in melanoma [37], glioblastoma [38], breast cancer [39], head and neck squamous cell carcinomas [40], and hepatocellular carcinoma [41]; however, and by contrast with our result, CSPG4 expression was associated with poor prognosis. By contrast, no prognostic value was identified in acute myeloid leukemias [42]. In sarcomas, two studies reported poor-prognostic value in chordoma [41] and soft tissue sarcomas [43]; although, in the latter, NG2/CSPG4 depletion showed divergent effects, depending on the developmental stage of sarcoma [44]. To our knowledge, no study has been reported in GIST. Functional studies are now warranted to address the functional role of CSPG4 expression in GIST, and notably, whether it is dependent on the link with the immune response we observed, and/or with direct influence on cancer cells.

CSPG4, a transmembrane proteoglycan, had been originally identified as a highly immunogenic tumor antigen on the surface of melanoma cells [45], and during the last decades, several works proposed it as a new therapeutic target for immune therapy in different cancers [25], including monoclonal antibodies in triple-negative breast cancer [46] and melanoma [47], antibody-drug conjugate in melanoma [48], and CAR-T cells in many cancers [49]. The recent study by Leuci et al. demonstrated in vitro and in vivo the anti-tumor activity of CSPG4-CAR.CIKs in soft tissue sarcomas, including GIST cell lines, and reported that tumor elimination in vitro was dependent on the expression level of tumor cells. This observation suggested that “CSPG4-high” GIST should represent the most adequate GIST to be targeted by such treatment. We thus compared their immune landscape, at the transcriptional level, with that of “CSPG4-low” GIST. We found significant variations between the two tumor groups, with higher expression of several immune variables in “CSPG4-high” tumors: (i) higher scores for immune signatures suggesting higher infiltration by immune cells; (ii) higher infiltrate in certain immune cell subtypes: DC, T cells, Tem, CD8 T cells, and numerous cytotoxic cells subsets, such as activated NK CD56^dim^ cells; (iii) higher expression of signatures evoking higher immune cytolytic activity. Altogether, these results suggested a higher anti-tumor immune response in “CSPG4-high” tumors than in “CSPG4-low” tumors that relies on both the adaptive and innate immune system. Of note, the Th1 immune cell type was not significantly different between the two tumor groups, suggesting that, even though the immune infiltrate was higher in “CSPG4-high” tumors, there is still room to increase anti-tumor immune response efficiency. The actual cytotoxic response seems to rely on innate NK-cell subset activation. This also has been suggested by several reports [20,50]. Of note, several studies describing immune features of clinical samples have already pointed out a role for NK cells in GIST. IHC analysis of 91 samples showed tumor infiltrated by a homogeneous subset of cytokine-secreting CD56^bright^ NK cells that accumulated in tumor foci after imatinib treatment [17], and the density of the NK infiltrate independently predicted the progression-free survival. Analysis of transcriptional data showed a higher activated NK signature in GIST than in three other sarcoma subtypes [51].

In this fertile immune environment, complementing the boost of NK cells with immune checkpoint inhibitors, which will fully unleash CD8 T cells cytotoxic potential, might be extremely powerful. Novel immunotherapies, such as anti-NKG2A or anti-KIR, that target both NK and other cytotoxic T cells subsets, might be promising as well. In this line, “CSPG4-high” GIST, as compared to “CSPG4-low” GIST, might be more sensitive to CSPG4-CAR.CIKs, not only because of higher target expression level, but also thanks to a more favorable anti-tumor cytokine microenvironment with a synergic action of resident cytotoxic T and NK cells. Such analysis has never been reported in GIST.

## 5. Conclusions

We showed that expression of CSPG4, a new potential target for immune therapy, notably specific CAR-CIKs, is heterogeneous in GIST clinical samples and that high expression is associated independently with DFS and with an immune landscape favorable to induce strong cytotoxic immune response after NK cell stimulation. Our study displays several strengths: (i) its originality, (ii) a relatively large size of series given the rarity of GIST, and (iii) the biological and clinical relevance of *CSPG4* expression. It also includes a few limitations: (i) its retrospective nature and (ii) the mRNA, rather than protein, analysis on bulk tissue samples. Of course, analysis of larger patient series is warranted to confirm our observation, as well as functional analyses of GIST preclinical models. However, our results suggest the potential value of CSPG4-CAR.CIKs treatment in GIST, notably “CSPG4-high” tumors, that calls for drug testing in vivo, and then in clinical trials.

## Figures and Tables

**Table 1:** Clinicopathological characteristics of patients and tumors.

Characteristics		N	All	*CSPG4 Group*		*p*-Value
				Low	High	
Age median (range), years		194	61 (8–87)	56.29 (18–84)	59.88 (8–87)	0.107
Sex		255				0.430
	female		110 (43%)	46 (40%)	64 (46%)	
	male		145 (57%)	69 (60%)	76 (54%)	
Mutation		275				0.063
	wild-type		44 (16%)	25 (20%)	19 (13%)	
	*KIT*		190 (69%)	90 (70%)	100 (68%)	
	*PDGFRA*		41 (15%)	13 (10%)	28 (19%)	
Site		242				4 × 10^−6^
	gastric		178 (74%)	61 (58%)	117 (86%)	
	small intestine		43 (18%)	30 (28%)	13 (10%)	
	other		21 (9%)	15 (14%)	6 (4%)	
AFIP risk		161				3.83 × 10^−4^
	low		85 (53%)	21 (33%)	64 (65%)	
	intermediate		31 (19%)	17 (27%)	14 (14%)	
	high		45 (28%)	25 (40%)	20 (20%)	
Extension stage						2.83 × 10^−3^
	advanced	39	39 (17%)	26 (26%)	13 (10%)	
	localized	187	187 (83%)	73 (74%)	114 (90%)	
Follow-up median, months (min-max)		87	46 (2–165)	44 (5–101)	45 (2–165)	0.904
DFS event, N (%)		161	32 (20%)	24 (39%)	8 (8%)	3.79 × 10^−6^
5-year DFS		87	79% (69–90)	59% (38–90)	90% (81–99)	1.15 × 10^−2^

**Table 2:** Uni- and multivariate analyses for disease-free survival.

Characteristics	Univariate			Multivariate		
	N	OR (95%CI)	*p*-Value	N	OR (95%CI)	*p*-Value
Age, years	101	1.01 (0.96–1.05)	0.796			
Sex, male vs. female	161	1.90 (0.82–4.43)	0.137			
Mutation, KIT vs. wild-type	160	2.83 (0.62–13.01)	0.181			
Mutation, PDGFRA vs. wild-type		0.35 (0.03–4.23)	0.412			
Site, other vs. gastric	161	2.35 (0.55–10.08)	0.251			
Site, small intestine vs. gastric		1.88 (0.66–5.36)	0.239			
AFIP risk, high vs. low/intermediate	160	26.48 (9.57–73.29)	2.82 × 10^−10^	160	26.04 (8.64–78.46)	6.93 × 10^−9^
CSPG4, high vs. low	161	0.14 (0.057–0.34)	1.26 × 10^−5^	160	0.14 (0.048–0.43)	5.12 × 10^−4^

## Data Availability

All data sets are publicly available, and references are described in Table S1.

## Supplementary Materials

The following supporting information can be downloaded at: https://www.mdpi.com/article/10.3390/cancers14051306/s1, Figure S1: CSPG4 expression in GIST samples and cancer cell lines; Table S1: List of GIST data sets included. Table S2. Clinicopathological characteristics of patients and tumors included in the prognostic analysis.

## References

[1] Corless C.L., Barnett C.M., Heinrich M.C. (2011). Gastrointestinal Stromal Tumours: Origin and Molecular Oncology. Nat. Rev. Cancer.

[2] Casali P.G., Blay J.Y., Abecassis N., Bajpai J., Bauer S., Biagini R., Bielack S., Bonvalot S., Boukovinas I., Bovee J.V.M.G. (2022). Gastrointestinal Stromal Tumours: ESMO-EURACAN-GENTURIS Clinical Practice Guidelines for Diagnosis, Treatment and Follow-Up. Ann. Oncol. Off. J. Eur. Soc. Med. Oncol..

[3] Hirota S., Isozaki K., Moriyama Y., Hashimoto K., Nishida T., Ishiguro S., Kawano K., Hanada M., Kurata A., Takeda M. (1998). Gain-of-Function Mutations of c-Kit in Human Gastrointestinal Stromal Tumors. Science.

[4] Heinrich M.C., Corless C.L., Duensing A., McGreevey L., Chen C.-J., Joseph N., Singer S., Griffith D.J., Haley A., Town A. (2003). PDGFRA Activating Mutations in Gastrointestinal Stromal Tumors. Science.

[5] Joensuu H., DeMatteo R.P. (2012). The Management of Gastrointestinal Stromal Tumors: A Model for Targeted and Multidisciplinary Therapy of Malignancy. Annu. Rev. Med..

[6] Antonescu C.R. (2011). The GIST Paradigm: Lessons for Other Kinase-Driven Cancers. J. Pathol..

[7] Dematteo R.P., Ballman K.V., Antonescu C.R., Maki R.G., Pisters P.W.T., Demetri G.D., Blackstein M.E., Blanke C.D., von Mehren M., Brennan M.F. (2009). Adjuvant Imatinib Mesylate after Resection of Localised, Primary Gastrointestinal Stromal Tumour: A Randomised, Double-Blind, Placebo-Controlled Trial. Lancet Lond. Engl..

[8] Joensuu H., Eriksson M., Sundby Hall K., Hartmann J.T., Pink D., Schütte J., Ramadori G., Hohenberger P., Duyster J., Al-Batran S.-E. (2012). One vs Three Years of Adjuvant Imatinib for Operable Gastrointestinal Stromal Tumor: A Randomized Trial. JAMA.

[9] Miettinen M., Lasota J. (2006). Gastrointestinal Stromal Tumors: Pathology and Prognosis at Different Sites. Semin. Diagn. Pathol..

[10] Gold J.S., Gönen M., Gutiérrez A., Broto J.M., García-del-Muro X., Smyrk T.C., Maki R.G., Singer S., Brennan M.F., Antonescu C.R. (2009). Development and Validation of a Prognostic Nomogram for Recurrence-Free Survival after Complete Surgical Resection of Localised Primary Gastrointestinal Stromal Tumour: A Retrospective Analysis. Lancet Oncol..

[11] Rossi S., Miceli R., Messerini L., Bearzi I., Mazzoleni G., Capella C., Arrigoni G., Sonzogni A., Sidoni A., Toffolatti L. (2011). Natural History of Imatinib-Naive GISTs: A Retrospective Analysis of 929 Cases with Long-Term Follow-up and Development of a Survival Nomogram Based on Mitotic Index and Size as Continuous Variables. Am. J. Surg. Pathol..

[12] Patel S. (2011). Navigating Risk Stratification Systems for the Management of Patients with GIST. Ann. Surg. Oncol..

[13] Wozniak A., Rutkowski P., Schöffski P., Ray-Coquard I., Hostein I., Schildhaus H.-U., Le Cesne A., Bylina E., Limon J., Blay J.-Y. (2014). Tumor Genotype Is an Independent Prognostic Factor in Primary Gastrointestinal Stromal Tumors of Gastric Origin: A European Multicenter Analysis Based on ConticaGIST. Clin. Cancer Res. Off. J. Am. Assoc. Cancer Res..

[14] Chantharasamee J., Adashek J.J., Wong K., Eckardt M.A., Chmielowski B., Dry S., Eilber F.C., Singh A.S. (2021). Translating Knowledge About the Immune Microenvironment of Gastrointestinal Stromal Tumors into Effective Clinical Strategies. Curr. Treat. Options Oncol..

[15] Balachandran V.P., Cavnar M.J., Zeng S., Bamboat Z.M., Ocuin L.M., Obaid H., Sorenson E.C., Popow R., Ariyan C., Rossi F. (2011). Imatinib Potentiates Antitumor T Cell Responses in Gastrointestinal Stromal Tumor through the Inhibition of Ido. Nat. Med..

[16] Cameron S., Haller F., Dudas J., Moriconi F., Gunawan B., Armbrust T., Langer C., Füzesi L., Ramadori G. (2008). Immune Cells in Primary Gastrointestinal Stromal Tumors. Eur. J. Gastroenterol. Hepatol..

[17] Rusakiewicz S., Semeraro M., Sarabi M., Desbois M., Locher C., Mendez R., Vimond N., Concha A., Garrido F., Isambert N. (2013). Immune Infiltrates Are Prognostic Factors in Localized Gastrointestinal Stromal Tumors. Cancer Res..

[18] Van Dongen M., Savage N.D.L., Jordanova E.S., Briaire-de Bruijn I.H., Walburg K.V., Ottenhoff T.H.M., Hogendoorn P.C.W., van der Burg S.H., Gelderblom H., van Hall T. (2010). Anti-Inflammatory M2 Type Macrophages Characterize Metastasized and Tyrosine Kinase Inhibitor-Treated Gastrointestinal Stromal Tumors. Int. J. Cancer.

[19] Ménard C., Blay J.-Y., Borg C., Michiels S., Ghiringhelli F., Robert C., Nonn C., Chaput N., Taïeb J., Delahaye N.F. (2009). Natural Killer Cell IFN-Gamma Levels Predict Long-Term Survival with Imatinib Mesylate Therapy in Gastrointestinal Stromal Tumor-Bearing Patients. Cancer Res..

[20] Delahaye N.F., Rusakiewicz S., Martins I., Ménard C., Roux S., Lyonnet L., Paul P., Sarabi M., Chaput N., Semeraro M. (2011). Alternatively Spliced NKp30 Isoforms Affect the Prognosis of Gastrointestinal Stromal Tumors. Nat. Med..

[21] Pantaleo M.A., Tarantino G., Agostinelli C., Urbini M., Nannini M., Saponara M., Castelli C., Stacchiotti S., Fumagalli E., Gatto L. (2019). Immune Microenvironment Profiling of Gastrointestinal Stromal Tumors (GIST) Shows Gene Expression Patterns Associated to Immune Checkpoint Inhibitors Response. Oncoimmunology.

[22] Borg C., Terme M., Taïeb J., Ménard C., Flament C., Robert C., Maruyama K., Wakasugi H., Angevin E., Thielemans K. (2004). Novel Mode of Action of C-Kit Tyrosine Kinase Inhibitors Leading to NK Cell-Dependent Antitumor Effects. J. Clin. Invest..

[23] Bertucci F., Finetti P., Mamessier E., Pantaleo M.A., Astolfi A., Ostrowski J., Birnbaum D. (2015). PDL1 Expression Is an Independent Prognostic Factor in Localized GIST. Oncoimmunology.

[24] Katz S.C., Burga R.A., Naheed S., Licata L.A., Thorn M., Osgood D., Nguyen C.T., Espat N.J., Fletcher J.A., Junghans R.P. (2013). Anti-KIT Designer T Cells for the Treatment of Gastrointestinal Stromal Tumor. J. Transl. Med..

[25] Ilieva K.M., Cheung A., Mele S., Chiaruttini G., Crescioli S., Griffin M., Nakamura M., Spicer J.F., Tsoka S., Lacy K.E. (2018). Chondroitin Sulfate Proteoglycan 4 and Its Potential As an Antibody Immunotherapy Target across Different Tumor Types. Front. Immunol..

[26] Leuci V., Donini C., Grignani G., Rotolo R., Mesiano G., Fiorino E., Gammaitoni L., D’Ambrosio L., Merlini A., Landoni E. (2020). CSPG4-Specific CAR.CIK Lymphocytes as a Novel Therapy for the Treatment of Multiple Soft-Tissue Sarcoma Histotypes. Clin. Cancer Res..

[27] Bindea G., Mlecnik B., Tosolini M., Kirilovsky A., Waldner M., Obenauf A.C., Angell H., Fredriksen T., Lafontaine L., Berger A. (2013). Spatiotemporal Dynamics of Intratumoral Immune Cells Reveal the Immune Landscape in Human Cancer. Immunity.

[28] Hendrickx W., Simeone I., Anjum S., Mokrab Y., Bertucci F., Finetti P., Curigliano G., Seliger B., Cerulo L., Tomei S. (2017). Identification of Genetic Determinants of Breast Cancer Immune Phenotypes by Integrative Genome-Scale Analysis. OncoImmunology.

[29] Ayers M., Lunceford J., Nebozhyn M., Murphy E., Loboda A., Kaufman D.R., Albright A., Cheng J.D., Kang S.P., Shankaran V. (2017). IFN-**γ**–Related MRNA Profile Predicts Clinical Response to PD-1 Blockade. J. Clin. Invest..

[30] Coppola D., Nebozhyn M., Khalil F., Dai H., Yeatman T., Loboda A., Mulé J.J. (2011). Unique Ectopic Lymph Node-like Structures Present in Human Primary Colorectal Carcinoma Are Identified by Immune Gene Array Profiling. Am. J. Pathol..

[31] Rooney M.S., Shukla S.A., Wu C.J., Getz G., Hacohen N. (2015). Molecular and Genetic Properties of Tumors Associated with Local Immune Cytolytic Activity. Cell.

[32] Gatza M.L., Lucas J.E., Barry W.T., Kim J.W., Wang Q., Crawford M.D., Datto M.B., Kelley M., Mathey-Prevot B., Potti A. (2010). A Pathway-Based Classification of Human Breast Cancer. Proc. Natl. Acad. Sci. USA.

[33] Thompson J.C., Davis C., Deshpande C., Hwang W.-T., Jeffries S., Huang A., Mitchell T.C., Langer C.J., Albelda S.M. (2020). Gene Signature of Antigen Processing and Presentation Machinery Predicts Response to Checkpoint Blockade in Non-Small Cell Lung Cancer (NSCLC) and Melanoma. J. Immunother. Cancer.

[34] Dai D., Xie L., Shui Y., Li J., Wei Q. (2021). Identification of Tumor Microenvironment-Related Prognostic Genes in Sarcoma. Front. Genet..

[35] Rink L., Skorobogatko Y., Kossenkov A.V., Belinsky M.G., Pajak T., Heinrich M.C., Blanke C.D., von Mehren M., Ochs M.F., Eisenberg B. (2009). Gene Expression Signatures and Response to Imatinib Mesylate in Gastrointestinal Stromal Tumor. Mol. Cancer Ther..

[36] Bertucci F., Finetti P., Simeone I., Hendrickx W., Wang E., Marincola F.M., Viens P., Mamessier E., Ceccarelli M., Birnbaum D. (2018). The Immunologic Constant of Rejection Classification Refines the Prognostic Value of Conventional Prognostic Signatures in Breast Cancer. Br. J. Cancer.

[37] Price M.A., Colvin Wanshura L.E., Yang J., Carlson J., Xiang B., Li G., Ferrone S., Dudek A.Z., Turley E.A., McCarthy J.B. (2011). CSPG4, a Potential Therapeutic Target, Facilitates Malignant Progression of Melanoma. Pigment Cell Melanoma Res..

[38] Svendsen A., Verhoeff J.J.C., Immervoll H., Brøgger J.C., Kmiecik J., Poli A., Netland I.A., Prestegarden L., Planagumà J., Torsvik A. (2011). Expression of the Progenitor Marker NG2/CSPG4 Predicts Poor Survival and Resistance to Ionising Radiation in Glioblastoma. Acta Neuropathol..

[39] Hsu N.C., Nien P.-Y., Yokoyama K.K., Chu P.-Y., Hou M.-F. (2013). High Chondroitin Sulfate Proteoglycan 4 Expression Correlates with Poor Outcome in Patients with Breast Cancer. Biochem. Biophys. Res. Commun..

[40] Warta R., Herold-Mende C., Chaisaingmongkol J., Popanda O., Mock A., Mogler C., Osswald F., Herpel E., Küstner S., Eckstein V. (2014). Reduced Promoter Methylation and Increased Expression of CSPG4 Negatively Influences Survival of HNSCC Patients. Int. J. Cancer.

[41] Lu L.-L., Sun J., Lai J.-J., Jiang Y., Bai L.-H., Zhang L.-D. (2015). Neuron-Glial Antigen 2 Overexpression in Hepatocellular Carcinoma Predicts Poor Prognosis. World J. Gastroenterol..

[42] Hoffmeister L.M., Orhan E., Walter C., Niktoreh N., Hanenberg H., von Neuhoff N., Reinhardt D., Schneider M. (2021). Impact of KMT2A Rearrangement and CSPG4 Expression in Pediatric Acute Myeloid Leukemia. Cancers.

[43] Benassi M.S., Pazzaglia L., Chiechi A., Alberghini M., Conti A., Cattaruzza S., Wassermann B., Picci P., Perris R. (2009). NG2 Expression Predicts the Metastasis Formation in Soft-Tissue Sarcoma Patients. J. Orthop. Res. Off. Publ. Orthop. Res. Soc..

[44] Hsu S.-H.C., Nadesan P., Puviindran V., Stallcup W.B., Kirsch D.G., Alman B.A. (2018). Effects of Chondroitin Sulfate Proteoglycan 4 (NG2/CSPG4) on Soft-Tissue Sarcoma Growth Depend on Tumor Developmental Stage. J. Biol. Chem..

[45] Wilson B.S., Imai K., Natali P.G., Ferrone S. (1981). Distribution and Molecular Characterization of a Cell-Surface and a Cytoplasmic Antigen Detectable in Human Melanoma Cells with Monoclonal Antibodies. Int. J. Cancer.

[46] Wang X., Osada T., Wang Y., Yu L., Sakakura K., Katayama A., McCarthy J.B., Brufsky A., Chivukula M., Khoury T. (2010). CSPG4 Protein as a New Target for the Antibody-Based Immunotherapy of Triple-Negative Breast Cancer. J. Natl. Cancer Inst..

[47] Uranowska K., Samadaei M., Kalic T., Pinter M., Breiteneder H., Hafner C. (2021). A Chondroitin Sulfate Proteoglycan 4-specific Monoclonal Antibody Inhibits Melanoma Cell Invasion in a Spheroid Model. Int. J. Oncol..

[48] Hoffmann R.M., Crescioli S., Mele S., Sachouli E., Cheung A., Chui C.K., Andriollo P., Jackson P.J.M., Lacy K.E., Spicer J.F. (2020). A Novel Antibody-Drug Conjugate (ADC) Delivering a DNA Mono-Alkylating Payload to Chondroitin Sulfate Proteoglycan (CSPG4)-Expressing Melanoma. Cancers.

[49] Harrer D.C., Dörrie J., Schaft N. (2019). CSPG4 as Target for CAR-T-Cell Therapy of Various Tumor Entities-Merits and Challenges. Int. J. Mol. Sci..

[50] Komita H., Koido S., Hayashi K., Kan S., Ito M., Kamata Y., Suzuki M., Homma S. (2015). Expression of Immune Checkpoint Molecules of T Cell Immunoglobulin and Mucin Protein 3/Galectin-9 for NK Cell Suppression in Human Gastrointestinal Stromal Tumors. Oncol. Rep..

[51] Dufresne A., Lesluyes T., Ménétrier-Caux C., Brahmi M., Darbo E., Toulmonde M., Italiano A., Mir O., Le Cesne A., Le Guellec S. (2020). Specific Immune Landscapes and Immune Checkpoint Expressions in Histotypes and Molecular Subtypes of Sarcoma. Oncoimmunology.

