# Supplementary material for: CSPG4 Expression in GIST Is Associated with Better Prognosis and Strong Cytotoxic Immune Response
Source: Cancers (Basel). 2022 Mar 3;14(5):1306. doi: 10.3390/cancers14051306 (PMC8909029; doi:10.3390/cancers14051306)
Supplement: Supplementary file 1 [file cancers-14-01306-s001.zip › cancers-1596013-supplementary.pdf]

cancers

MDPI

Supplementary Materials: CSPG4 expression in GIST is associated with better prognosis and strong cytotoxic immune response

Alexandre de Nonneville, Pascal Finetti, Maelle Picard, Audrey Monneur, Maria Abbondanza Pantaleo, Annalisa Astolfi, Jerzy Ostrowski, Daniel Birnbaum, Emilie Mamessier and François Bertucci

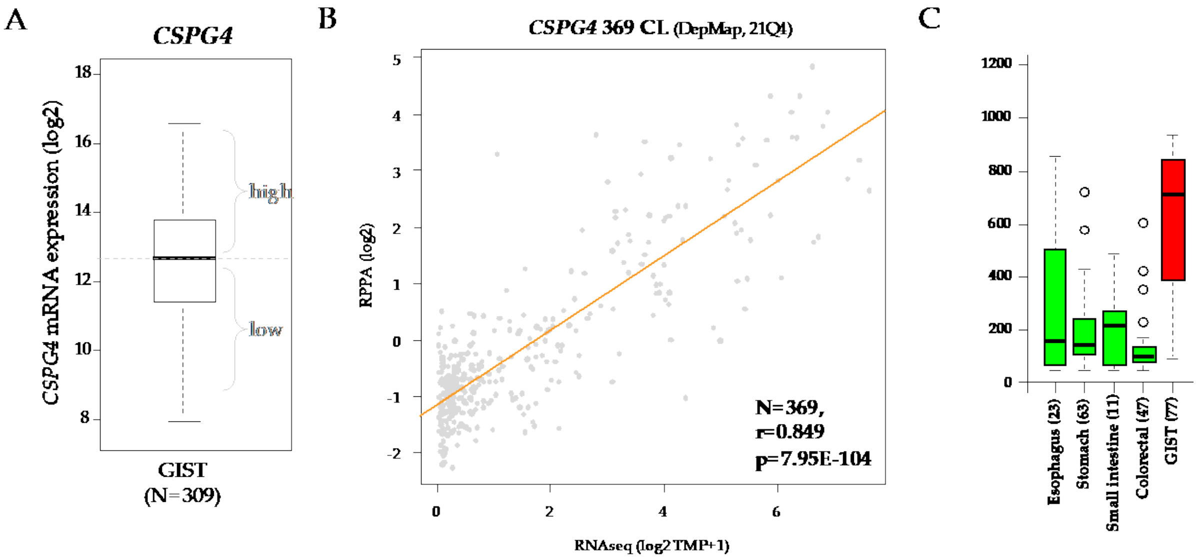

Figure S1. CSPG4 expression in GIST samples and cancer cell lines. (**A**) Box plot of mRNA expression levels in the 309 GIST clinical samples. (**B**) Pearson **c**orrelation between *CSPG4* mRNA (RNAseq data) and protein (RPPA) expression in 369 cancer cell lines. C/ *CSPG4* mRNA expression in GIST samples and digestive tract normal tissues according to IST Online (access date: October 20, 2020).

Table S1. List of GIST data sets included

Reference	Source of data	Technological platform	N° of probe sets/genes	All samples	GIST samples included in the present analysis with CSPG4
Nielsen et al., J Clin Oncol 2002	GEO : GSE3443	Stanford spotted DNA	24K	46	9
Subramanian et al., Oncogene 2004	GEO : GSE4001	Stanford spotted DNA	41K	26	26
Baird et al., Cancer Res 2005	GEO : GSE2553	NHGRI cDNA Custom Array	12K	181	5
Detwiller et al., Cancer Res 2005	GEO : GSE2719	Affymetrix	22K	54	2
Kang et al., Oncogene 2005	http://www.molpathol.org/data/GIST-array-data.txt	Compugene	18K	22	22
West et al., PLoS Biology 2005	http://microarray-pubs.stanford.edu/	Stanford spotted DNA	42K	57	5
Yamaguchi et al., J Clin Oncol 2008	GEO : GSE8167	Affymetrix	54K	32	32
Ostrowski et al., BMC Cancer 2009	GEO : GSE17743	Affymetrix	54K	29	29
Rink et al., Mol Cancer Ther 2009	GEO : GSE15966	Agilent	44K	54	29
Astolfi et al., Lab Invest 2010	GEO : GSE20710	Affymetrix	54K	22	22
Hadju et al., J Pathol 2010	http://cbio.mskcc.org/public/SFT/	Affymetrix	54K	56	3
Yang et al., Technol Cancer Res Treat. 2010	http://www3.mdanderson.org/~genomics/sarcoma_data_matrix_for_supplemental.zip	Agilent	44K	68	37
Lagarde et al., Clin Cancer Res 2011	ArrayExpress : E-MTAB-373	Agilent	44K	60	60
Arne et al., Int J Cancer 2011	GEO : GSE14755	Swegene	55K	21	13
Lee et al., PLoS One 2013	GEO : GSE47911	Agilent	44K	16	15

Table S2. Clinicopathological characteristics of patients and tumors included in the prognostic analysis.

Characteristics	N	All	*CSPG4 group* low	*CSPG4 group* high	p-value
**Age median (range), years**	**101**	**62 (26-85)**	**59 (26-82)**	62 (40-85)	0.215
Sex	161				0.170
female		64 (40%)	20 (32%)	44 (44%)	
male		97 (60%)	42 (68%)	55 (56%)	
Mutation	160				2.56×10^{-2}
wild-type		19 (12%)	10 (16%)	9 (9%)	
KIT		116 (72%)	48 (77%)	68 (69%)	
PDGFRA		25 (16%)	4 (6%)	21 (21%)	
Site	161				1.49×10^{-6}
gastric		131 (81%)	38 (61%)	93 (94%)	
small intestine		21 (13%)	17 (27%)	4 (4%)	
other		9 (6%)	7 (11%)	2 (2%)	
AFIP risk	160				4.82×10^{-4}
low		85 (53%)	21 (34%)	64 (65%)	
intermediate		31 (19%)	16 (26%)	15 (15%)	
high		44 (28%)	25 (40%)	19 (19%)	
Extension stage	161				NA
advanced		0 (0%)	0 (0%)	0 (0%)	
localized		161 (100%)	62 (100%)	99 (100%)	
Follow-up median, months (min-max)	87	46 (2-165)	44 (5-101)	45 (2-165)	0.904
DFS event, N (%)	161	32 (20%)	24 (39%)	8 (8%)	3.79×10^{-6}
5-year DFS	87	79% [69-90]	59% [38-90]	90% [81-99]	1.15×10^{-2}
